# Translating innovation into impact: a national policy dialogue on scaling and integrating evidence-based supportive text messaging into Canada's mental health care systems

**DOI:** 10.3389/fdgth.2026.1830295

**Published:** 2026-07-27

**Authors:** Belinda Agyapong, Linda Liebenberg, Ejemai Eboreime, Andrew J. Greenshaw, Vincent I. O. Agyapong

**Affiliations:** 1 Department of Psychiatry, Dalhousie University, Halifax, NS, Canada; 2 Dalhousie Centre for Global Mental Health, Dalhousie University, Halifax, NS, Canada; 3 Department of Psychiatry, University of Alberta, Edmonton, AB, Canada

**Keywords:** Canada, digital mental health, implementation, policy dialogue, supportive text messaging

## Abstract

**Background:**

Supportive text messaging interventions have demonstrated efficacy in reducing symptoms of depression, anxiety, and stress, yet their integration into Canadian mental health systems remains variable. This national policy dialogue examined governance, implementation, and equity considerations associated with scaling evidence-based supportive text messaging programs across provincial and territorial care systems.

**Methods:**

A structured virtual policy dialogue convened senior representatives from provincial and territorial health authorities, federal agencies, community mental health organizations, crisis service providers, philanthropic foundations, and academic researchers. The session combined an evidence review with moderated deliberation, focusing on system integration, crisis pathway alignment, funding models, digital infrastructure, equity, and evaluation. The dialogue was transcribed verbatim and analyzed using an inductive–deductive thematic analysis. Interpretation of findings was informed by the Consolidated Framework for Implementation Research (CFIR), the Exploration, Preparation, Implementation and Sustainment (EPIS) framework, and the Reach, Effectiveness, Adoption, Implementation, and Maintenance (RE-AIM) framework to examine implementation determinants, scalability, sustainability, and health system integration.

**Results:**

Stakeholders demonstrated broad consensus regarding the scalability, feasibility, and cost-effectiveness of supportive text messaging. High-priority integration points included post-emergency department discharge, post-psychiatric hospitalization follow-up, suicide prevention caring contact models, and stepped-care pathways. Participants emphasized the need to embed digital interventions within existing community mental health infrastructure rather than displacing in-person services. Equity considerations, including cellular connectivity limitations in the northern regions, linguistic diversity, and Indigenous co-creation, were central to implementation discussions. Fragmentation across jurisdictions and inconsistent evaluation frameworks were identified as barriers to coordinated scale-up. There was support for exploring pan-Canadian coordination mechanisms while preserving provincial autonomy.

**Conclusions:**

Supportive text messaging represents a mature, evidence-based digital intervention with potential to enhance continuity of care and optimize system capacity. The primary barriers to broader integration appear structural rather than scientific. Coordinated implementation, sustained funding, culturally grounded co-design, and standardized evaluation frameworks will be essential to realizing its system-level impact within Canada's mental health care landscape.

## 1 Introduction

Canada's mental health and substance use burden continues to outpace system capacity, with persistent gaps in timely access to evidence-based psychosocial care across the continuum; from prevention and early intervention to crisis response and post-discharge follow-up (1). Digital mental health interventions (2–5) are increasingly positioned as one lever to expand reach, reduce geographic barriers, and augment constrained workforces (6); however, integration into routine care remains uneven due to variability in governance, procurement, privacy requirements, interoperability, and sustainable financing across provinces and territories.

Implementation research consistently demonstrates that successful integration of digital mental health interventions depends not only on clinical effectiveness but also on addressing multilevel determinants operating at the policy, organizational, provider, intervention, and user levels (7, 8). Common barriers include fragmented governance, inadequate funding mechanisms, limited interoperability, clinician readiness, digital literacy, privacy concerns, and challenges sustaining implementation beyond initial adoption (8). Frameworks such as the Consolidated Framework for Implementation Research (CFIR) (9, 10), the Exploration, Preparation, Implementation and Sustainment (EPIS) framework (11), and the Reach, Effectiveness, Adoption, Implementation and Maintenance (RE-AIM) framework (12) have been widely applied to understand and address these implementation challenges across health systems (8, 13). Within this landscape, short message service (SMS)-based supportive messaging represents a pragmatic, low-barrier modality that can be delivered at population scale without smartphones, data plans, or app installation. SMS-based interventions have demonstrated high acceptability, feasibility, and scalability across a range of health conditions, including chronic disease management, medication adherence, smoking cessation, and mental health. Within mental health and addictions, randomized controlled trials (14–17) and systematic reviews (18–21) have shown that supportive text messaging can reduce symptoms of depression, anxiety, stress, alcohol and drug use and psychological distress while improving resilience and patient satisfaction. In addition, emerging economic evaluations indicate that supportive text messaging is highly scalable and can reduce subsequent health service utilization, supporting its potential as a cost-effective adjunct to routine mental health care (4). These findings suggest that supportive text messaging has progressed beyond proof-of-concept and represents a mature evidence-informed intervention suitable for broader health system implementation and policy consideration. Nevertheless, systematic reviews report considerable heterogeneity in intervention content, frequency, duration, theoretical underpinning, participant populations, and methodological quality, highlighting the importance of rigorous evaluation and context-specific implementation when scaling these interventions (19–22). Importantly, supportive messaging can be conceptualized as a “micro-intervention”: brief, frequent prompts that reinforce coping skills (often drawing on cognitive-behavioural and related approaches), normalize distress, and encourage self-management-mechanisms that may be particularly relevant when formal care is delayed or episodic.

In parallel, Canada is strengthening crisis response infrastructure through national initiatives such as the 9-8-8: Suicide Crisis Helpline, launched in November 2023, with early implementation described using a public health and implementation framework lens (23, 24). A key policy question is how to connect crisis services to scalable “care after the call/text,” including brief interventions that can support stabilization, continuity, and linkage to local resources. Evidence from suicide prevention also supports the utility of low-intensity, post-contact outreach approaches (e.g., caring contacts), reinforcing the plausibility of structured, brief follow-up modalities as part of a stepped-care strategy (25).

Youth mental health system transformation further strengthens the case for low-barrier digital supports embedded within integrated pathways. Canadian youth service initiatives emphasize timely, acceptable access options and implementation structures that can support scale, spread, and co-designed digital components (26). Co-creation with youth, Indigenous communities, and other equity-deserving groups is increasingly recognized as essential for cultural safety, relevance, and sustained engagement (27, 28), particularly where digital interventions risk exacerbating inequities if language, context, and access realities are not addressed from inception (29, 30). Against this backdrop, a National Policy Dialogue, entitled “Translating Innovation into Impact: Integrating Digital Mental Health into Canada's Care Systems” convened senior leaders from federal, provincial, and territorial governments, health authorities, philanthropic organizations, community mental health agencies, integrated youth service organizations, academia, and the digital health sector. The dialogue sought to examine the opportunities, challenges, and policy considerations associated with integrating evidence-based supportive text messaging into Canadian mental health systems. Supportive text messaging was used as a scalable, evidence-informed, and policy-ready case study to explore broader issues related to implementation, governance, equity, sustainability, and national coordination of digital mental health innovations. The Dialogue also explored complementary digital functions (e.g., AI-assisted navigation tools using curated resources) and the governance, evaluation, and sustainability conditions required for national coordination while preserving provincial/territorial adaptability. This manuscript describes the rationale for national integration of supportive text messaging within a stepped-care digital mental health strategy, and to report policy-relevant themes arising from a pan-Canadian policy dialogue focused on implementation, equity, evaluation, and system integration across prevention, crisis pathways, and post-discharge/community care.

## 2 Methods

### 2.1 Study design

This study employed a qualitative descriptive design using a structured national policy dialogue as the primary data source. Policy dialogues are recognized as a knowledge translation approach that facilitates deliberative exchange among stakeholders and supports the integration of research evidence into policy and system decision-making (31, 32). The dialogue was convened to examine the system-level integration of evidence-based supportive text messaging programs within Canadian mental health care systems. Participants represented federal organizations as well as provincial and territorial governments, health authorities, and partner organizations from Alberta, British Columbia, Saskatchewan, Manitoba, New Brunswick, Nova Scotia, Yukon and the Northwest Territories. This broad representation enabled discussion of implementation considerations across diverse health system contexts, including urban, rural, remote, and northern settings.

The session combined evidence presentation with structured, moderated stakeholder discussion. The analytic objective was to identify system-level themes related to implementation, governance, equity, and sustainability.

### 2.2 Setting and participants

Participants were purposively invited to ensure representation across provincial and territorial health authorities, federal agencies, community mental health organizations, crisis service organizations (e.g., 9-8-8, 211), philanthropic foundations, integrated youth service organizations, digital health innovators and academic researchers. Participants were recruited using maximum variation purposive sampling, a qualitative sampling strategy that intentionally seeks participants with diverse characteristics and experiences to capture a broad range of perspectives on the phenomenon of interest rather than statistical representativeness (33). This approach was used to ensure representation across federal, provincial, and territorial organizations, multiple governance levels, service sectors, and diverse geographic contexts, including northern, remote, rural, and urban jurisdictions. By incorporating variation in organizational roles and health system contexts, the dialogue sought to identify both common priorities and context-specific implementation considerations for integrating supportive text messaging into Canadian mental health systems. Participants included assistant deputy ministers, directors of federal organizations, health authority executives, crisis service leaders, community organization directors, foundation executives, and academic investigators. The dialogue was conducted virtually to facilitate national participation.

### 2.3 Dialogue structure

The session was structured into three components:

#### 2.3.1 Evidence review

Presentation of the current evidence base regarding supportive text messaging interventions, including randomized controlled trials, cost-effectiveness findings, and implementation experiences.

#### 2.3.2 Moderated roundtable discussion

The moderated roundtable constituted the central deliberative component of the policy dialogue. An experienced academic facilitator with expertise in digital mental health guided participants through a structured discussion focused on implementation and policy-relevant domains. The discussion framework was developed in advance to ensure systematic exploration of key system-level considerations. Participants were invited to examine potential system integration points, including how supportive text messaging might be embedded within existing care pathways such as emergency department discharge, inpatient transitions, community mental health services, and navigation systems. Crisis pathway alignment was also explored, including relationships with national and provincial services (e.g., 9-8-8, 211, and 811) and the integration of asynchronous messaging within broader crisis response models. Additional discussion domains included governance and funding structures, jurisdictional roles, procurement considerations, and sustainability planning. Equity and co-creation were incorporated as core analytic lenses, with prompts addressing culturally grounded design, engagement with Indigenous and youth communities, and infrastructure variability in northern and remote contexts. The session also addressed evaluation frameworks, accountability mechanisms, digital infrastructure requirements, interoperability, and privacy considerations relevant to scalable implementation.

### 2.4 Forward-looking deliberation

Participants were invited to articulate potential next steps for provincial, territorial, and national coordination.

### 2.5 Data collection

The full session was recorded and transcribed verbatim. Field notes were taken during the dialogue to capture contextual elements, including participant roles and points of emphasis. No structured interview guide was used; however, the moderator followed a pre-specified thematic framework informed by two complementary implementation science frameworks: the EPIS (11) framework, which examines factors influencing implementation across the stages of adoption and sustainment within outer and inner system contexts, and the RE-AIM (12) framework, which evaluates the public health impact, implementation, and sustainability of evidence-based interventions. These frameworks were selected because they provide complementary perspectives on implementation processes, organizational readiness, system integration, scalability, and long-term sustainability. The discussion was organized around policy-relevant domains including system integration, crisis pathway alignment, governance and funding, equity and co-design, evaluation and accountability, and digital infrastructure. The verbatim transcript constituted the primary qualitative dataset for analysis.

#### 2.5.1 Analytic approach

A thematic analysis approach was employed (34, 35). Analysis followed six iterative stages (36): (1) familiarization with the transcript; (2) initial open coding of policy-relevant concepts; (3) development of higher-order categories; (4) refinement of themes through constant comparison, whereby codes, emerging categories, and stakeholder perspectives were continuously compared within and across participant groups to identify similarities, differences, and relationships and to ensure that themes remained grounded in the data; (5) cross-sector validation of thematic coherence; and (6) synthesis into policy-relevant domains informed by the EPIS and RE-AIM implementation science frameworks.

Coding was conducted manually using an inductive–deductive hybrid strategy. Predefined sensitizing constructs (e.g., implementation, equity, governance, cost-effectiveness) informed deductive coding, while emergent concepts (e.g., digital fragmentation, capacity amplification, privacy–accountability tension) were captured inductively.

The analysis prioritized system-level themes rather than individual participant positions. Divergent perspectives were noted where present.

#### 2.5.2 Rigor and trustworthiness

To enhance trustworthiness, several strategies were incorporated throughout the analytic process. Triangulation was achieved by including participants from multiple governance levels and service sectors, thereby reducing the risk of single-sector bias. Reflexivity was maintained by explicitly acknowledging the facilitation team's professional backgrounds in digital mental health research and policy, and by reflecting on how these perspectives might shape interpretation. An audit trail was developed through systematic documentation of coding decisions and theme refinement. Finally, preliminary thematic syntheses were reviewed within the analytic team to ensure internal coherence and alignment with the original transcript data. Given the policy dialogue format, member checking with individual participants was not formally conducted. However, the structured format allowed real-time clarification of positions during the session.

### 2.6 Ethical considerations

The dialogue was conducted as a policy consultation and knowledge translation activity rather than human subjects research involving patients or personal health information. Participants engaged in their professional capacities. No individual-level identifying analysis was performed, and findings are reported at the thematic level.

Institutional research ethics board review was not required under Canadian Tri-Council guidelines for professional stakeholder consultation activities focused on policy deliberation (37). Nonetheless, confidentiality of discussion content was respected in reporting.

### 2.7 Positionality

The convening and analytic team included researchers with longstanding involvement in digital mental health evaluation. This positionality may have influenced emphasis on evidence-based interventions and implementation science frameworks. Efforts were made to represent both supportive and cautionary perspectives expressed during the dialogue.

## 3 Results

### 3.1 Participant representation

The National Policy Dialogue convened representatives from provincial and territorial health authorities, federal agencies, community mental health organizations, philanthropic foundations, digital health innovators, and academic researchers. Participants represented multiple jurisdictions across Canada, including northern and remote regions. Stakeholders included senior system leaders, assistant deputy ministers, crisis service coordinators, integrated youth service leads, and non-profit mental health providers.

The discussion focused on integration, governance, sustainability, and scale-up of evidence-based supportive text messaging programs within Canada's mental health systems.

Thematic analysis of the transcript identified eight major domains.

### 3.2 Transition from innovation to system integration

Participants consistently characterized supportive text messaging as a mature intervention supported by randomized controlled trials and cost-effectiveness data. The discussion shifted from efficacy to implementation. Stakeholders emphasized that the central policy question was no longer whether the intervention works, but how it should be embedded into care pathways. Several jurisdictions noted prior pilot experience and expressed readiness to consider broader integration. Supportive text messaging was viewed as particularly suited for system-level integration due to low marginal delivery cost, scalability through existing telecommunications infrastructure, minimal clinical workflow disruption, and high user acceptability. Additionally, participants noted that one-way text message-based interventions may present fewer privacy and security concerns compared with many other digital health approaches, including mobile applications, web-based self-management platforms, patient portals, and AI-enabled conversational systems that typically require user accounts, continuous internet connectivity, and the collection, storage, or processing of substantial amounts of personal health information. They observed that text messaging typically involves simpler message delivery through existing telecommunications networks and requires minimal user data collection, which may reduce potential vulnerabilities related to data storage, third-party platforms, and complex application infrastructures. Participants suggested that these characteristics may facilitate implementation within publicly funded health systems while reducing some of the governance and interoperability challenges associated with more technologically complex digital interventions.

#### 3.2.1 High-yield integration points within the continuum of care

Participants identified specific clinical and system transition points where supportive text messaging could be operationalized, including suicide prevention “caring contact” models, crisis line follow-up (e.g., 9-8-8), community-based stepped-care programs, post-emergency department discharge, and post–psychiatric inpatient discharge. Health system leaders emphasized that hospital readmission reduction and emergency department diversion are priority targets. The intervention was seen as a potential bridge between acute stabilization and longer-term care engagement. Several jurisdictions described ongoing reforms focused on front-door consolidation (e.g., 211, 811, 9-8-8 integration), noting that supportive text messaging could complement these access systems.

### 3.3 Cost-effectiveness and system value

Cost-effectiveness emerged as a central theme. Participants referenced evidence demonstrating reductions in post-discharge health service utilization, including lower readmission rates and decreased acute care costs. The relatively low per-person cost of supportive text messaging was contrasted with the higher cost of inpatient and specialist services. Health authority leaders noted that scalable digital supports may allow systems to extend reach without proportional workforce expansion. However, stakeholders emphasized that digital interventions should not displace essential in-person services but rather optimize resource allocation.

### 3.4 Role of community mental health organizations

There was broad agreement that community mental health organizations are critical components of Canada's care ecosystem. Participants highlighted persistent underfunding of community agencies, overreliance on emergency departments, and fragmentation between clinical and community sectors. Supportive text messaging was described as a tool that could be deployed through community organizations, enhancing capacity without substantially increasing operational burden. Some participants emphasized the need to formally integrate community partners into digital health planning rather than treating them as downstream referral destinations.

### 3.5 Equity considerations and northern realities

Representatives from northern and remote jurisdictions described unique implementation challenges, including limited broadband infrastructure, multiple cellular carriers with uneven geographic coverage, prepaid phone plans, and overcrowded housing limiting privacy for voice calls. In this context, SMS-based delivery was viewed as advantageous due to lower bandwidth requirements, greater discretion than voice services and compatibility with basic mobile devices.

Participants also highlighted linguistic diversity, including Indigenous dialect variability, as a critical implementation consideration.

#### 3.5.1 Co-creation and cultural adaptation

Stakeholders emphasized that successful digital implementation requires meaningful co-creation. Youth-serving organizations described structured co-design processes involving diverse youth participants, reporting improved engagement and acceptability. The distinction between *post hoc* content adaptation and true co-creation from problem definition through intervention design was discussed. Indigenous engagement was identified as particularly important, with participants noting that interventions must be locally contextualized. There was recognition that digital interventions may require regular content updating to maintain relevance, particularly among youth populations.

### 3.6 Evaluation, accountability, and data governance

Participants raised concerns regarding outcome measurement across mental health services more broadly. Several noted that many traditional services lack systematic outcome evaluation. In contrast, supportive text messaging was described as unusual in having multiple randomized trials and economic evaluations. However, tension was identified between anonymous service models and longitudinal outcome tracking. Stakeholders emphasized the importance of standardized outcome measures, embedded evaluation frameworks, privacy-respecting data collection models and transparent reporting. The need to balance confidentiality with accountability was discussed as an ongoing policy challenge.

### 3.7 Fragmentation and the case for pan-Canadian coordination

A recurring theme was digital fragmentation across provinces and territories. Participants described siloed innovation, variable funding cycles, and inconsistent implementation capacity. Several stakeholders proposed exploring a coordinated national approach, suggesting that shared infrastructure could reduce duplication, improve efficiency, support equitable access, and allow provincial customization within a common framework. While no formal agreement was reached, there was broad acknowledgement that pan-Canadian coordination warrants further exploration.

### 3.8 Summary of findings

The dialogue demonstrated a notable degree of cross-jurisdictional alignment regarding the role of supportive text messaging within Canada's mental health system. Participants consistently characterized supportive text messaging as an evidence-based and scalable intervention capable of reaching large populations with minimal operational burden. There was broad agreement that key transition points in care, particularly following emergency department visits, psychiatric hospitalization, and crisis service contact, represent high-impact opportunities for integration. Cost-effectiveness and overall system value were repeatedly identified as central considerations for policymakers and health system leaders. Stakeholders emphasized that scalable digital interventions must demonstrate not only clinical benefit but also measurable contributions to system efficiency and capacity. The discussion also underscored the importance of formally incorporating community mental health organizations into digital strategies. These organizations were recognized as critical partners in implementation, outreach, and culturally responsive adaptation. Equity considerations featured prominently, with attention drawn to infrastructure limitations in northern and remote regions, linguistic diversity, and the need for meaningful co-design with Indigenous communities, youth, and other equity-deserving populations.

Participants further emphasized that ongoing evaluation and accountability mechanisms are essential to long-term sustainability. While supportive text messaging was viewed positively across sectors, questions remain regarding optimal governance structures, funding pathways, evaluation frameworks, and mechanisms for coordinated interjurisdictional implementation. Overall, no substantive disagreement emerged about the intervention's potential role; rather, the remaining deliberations concern how best to structure and sustain its integration within Canada's mental health systems.

## 4 Discussion

This national policy dialogue examined the integration of evidence-based supportive text messaging interventions within Canada's mental health care systems. The findings reflect a transition in discourse from questions of efficacy toward questions of governance, sustainability, and system integration. Across jurisdictions, stakeholders demonstrated convergence on the potential role of scalable, low-cost digital interventions within stepped-care mental health models.

### 4.1 From efficacy to implementation science

The extensive body of evidence, including randomized controlled trials, systematic reviews, implementation studies, and economic evaluations supporting supportive text messaging interventions (6, 14–16, 38–41), as summarized in the Introduction, suggests that the field has progressed beyond demonstrating clinical efficacy toward addressing questions of implementation, scale-up, and sustainability. Consistent with this evolution, participants in the National Policy Dialogue emphasized that the principal policy challenge is no longer whether supportive text messaging is effective, but rather how evidence-based interventions can be systematically integrated into routine mental health care across diverse Canadian jurisdictions. This shift reflects broader implementation science principles, which recognize that successful translation of evidence into practice depends not only on intervention effectiveness but also on organizational readiness, governance, financing, stakeholder engagement, and long-term sustainability (11).

### 4.2 Strategic integration points within the continuum of care

Stakeholders consistently identified high-risk transition periods, particularly post-emergency department discharge and post–psychiatric hospitalization as priority integration points. The post-discharge period is well documented as a time of elevated suicide risk and system vulnerability (42, 43). Evidence-based “caring contacts” interventions have demonstrated reductions in suicide mortality and attempts when brief follow-up messages are delivered after clinical encounters (44, 45). Supportive text messaging was viewed as an operationally feasible extension of this evidence base, offering asynchronous continuity of care without imposing significant workforce burden. Importantly, participants emphasized that such interventions should function within stepped-care frameworks rather than replace higher-intensity services. Stepped-care approaches, which allocate resources proportionate to clinical need, are increasingly endorsed in Canadian and international mental health policy (46).

### 4.3 Cost-effectiveness and capacity amplification

Economic sustainability emerged as a central theme. Mental health systems in Canada face persistent workforce shortages (47, 48), long wait times, and rising demand (49). Digital interventions that demonstrate both clinical impact and downstream cost savings may offer a mechanism for system optimization. SMS-based interventions are characterized by low marginal costs and high scalability (40). Emerging economic analyses suggest that structured post-discharge messaging may reduce health service utilization and readmissions (4). These findings align with broader digital health literature demonstrating that remote monitoring and structured follow-up can reduce acute care demand (50). However, participants cautioned against framing digital tools as substitutes for relational or community-based services. Instead, supportive text messaging was conceptualized as a “capacity amplifier”, extending reach while preserving in-person services for individuals with higher acuity.

### 4.4 Community mental health and system fragmentation

The dialogue revealed ongoing concerns regarding overreliance on hospital-based services and underinvestment in community mental health organizations. Canadian mental health system analyses have repeatedly identified fragmentation between hospital, primary care, and community sectors (51). Digital interventions may serve as connective infrastructure between these sectors, particularly when embedded in navigation systems such as 9-8-8 and 211 services. However, the absence of standardized interjurisdictional coordination was identified as a barrier. Participants noted parallel digital initiatives occurring across provinces and territories, raising concerns about duplication and inefficiency. International experience suggests that coordinated national frameworks may improve digital health integration, provided they allow for local adaptation (52). Whether Canada should pursue a pan-Canadian digital mental health infrastructure remains a policy question requiring further deliberation.

### 4.5 Equity, cultural safety, and co-creation

Equity considerations were prominent throughout the dialogue. Northern and remote jurisdictions described infrastructure limitations, including variable broadband access and reliance on prepaid cellular services. SMS-based interventions may mitigate some aspects of the digital divide, given their low bandwidth requirements. Stakeholders emphasized the importance of meaningful co-creation, particularly with youth and Indigenous communities. Evidence from youth mental health services suggests that participatory co-design improves engagement and acceptability of digital interventions (53, 54). In Indigenous contexts, culturally grounded approaches and community leadership are essential to ensuring alignment with local knowledge systems and relational models of care (55). The distinction between superficial content tailoring and authentic co-development was repeatedly underscored. Implementation science literature similarly emphasizes that intervention fidelity and local adaptation must be balanced to achieve both effectiveness and cultural safety (13).

### 4.6 Evaluation, privacy, and accountability

The dialogue also highlighted tensions between anonymity and outcome evaluation. While crisis services prioritize confidentiality, long-term system accountability requires measurable outcomes. Notably, many traditional mental health services lack routine outcome monitoring, despite growing calls for measurement-based care (56). Supportive text messaging programs are relatively uncommon in having multiple randomized evaluations and economic analyses (14–16, 38, 39). Nonetheless, scaling such interventions nationally will require privacy-respecting evaluation frameworks capable of assessing population-level impact. Transparent reporting and standardized performance indicators will be critical to sustaining trust among policymakers and service users.

### 4.7 Limitations

This study has several limitations. First, although the dialogue included participants from multiple Canadian provinces and territories, federal agencies, health authorities, community organizations, philanthropic foundations, and academic institutions, not all provinces and territories were directly represented. Consequently, the findings may not fully capture jurisdiction-specific priorities, governance arrangements, or implementation challenges across all Canadian health systems. Nevertheless, the inclusion of federal organizations and national stakeholder groups, together with representation from diverse urban, rural, northern, and remote settings, provided a broad cross-jurisdictional perspective on policy considerations for scaling supportive text messaging. Future dialogues should seek participation from all provinces and territories to further enhance the comprehensiveness and transferability of the findings. Second, the findings reflect the perspectives of stakeholders participating in a national policy dialogue rather than empirical patient-level data or direct public consultation. Although participants represented multiple sectors, governance levels, and jurisdictions, their perspectives may not fully capture the experiences, priorities, or preferences of all patients, caregivers, Indigenous communities, or the broader public. Future implementation research should incorporate patient and public involvement alongside policy-level stakeholder engagement to ensure that digital mental health strategies remain responsive to the needs of end users. Third, although the EPIS and RE-AIM implementation science frameworks provided complementary and well-established approaches for examining implementation processes, system readiness, scalability, and sustainability, they inevitably shaped the organization and interpretation of the findings. These frameworks primarily emphasize implementation determinants, adoption, organizational context, and maintenance of evidence-based interventions, and may be less sensitive to broader sociopolitical influences, evolving policy environments, and emergent stakeholder dynamics that can also influence health system change. To minimize this risk, the analysis adopted an inductive–deductive approach in which themes were first identified directly from participants' discussions before being interpreted through the lens of the implementation science frameworks. This approach sought to preserve the richness of stakeholder perspectives while providing a structured framework for translating findings into policy-relevant implementation recommendations. Finally, as with all qualitative policy dialogues, the findings are intended to generate transferable policy insights rather than statistically generalizable conclusions. However, the diversity of participants and the consistency of themes across sectors strengthen the credibility and policy relevance of the findings.

### 4.8 Implications for Canadian mental health policy

The policy dialogue underscores the need to shift the focus of digital mental health from proof-of-concept toward sustained implementation within real-world systems. While continued clinical trials remain important, particularly for emerging technologies, participants emphasized that implementation research must proceed in parallel. Understanding how evidence-based digital interventions can be operationalized within provincial and territorial care pathways while maintaining fidelity, acceptability, and cost-effectiveness will be critical to achieving system-level impact. Transition points in care emerged as especially strategic opportunities for integration. Periods following emergency department visits, psychiatric hospitalization, and crisis service contact were identified as high-risk intervals in which low-intensity, scalable follow-up supports may strengthen continuity and reduce relapse or readmission. Embedding supportive digital interventions within discharge workflows and crisis pathways may offer a pragmatic means of extending care beyond episodic encounters. Participants also highlighted the importance of situating digital supports within stepped-care models. Digital interventions should not be conceptualized as replacements for in-person services, but rather as complementary layers that match intervention intensity to clinical need. Such integration may allow specialized and higher-intensity resources to be reserved for individuals with greater complexity, thereby improving overall system efficiency. The dialogue further reinforced that community mental health organizations must be recognized as formal partners in digital integration strategies. These organizations often serve as frontline access points and are well positioned to facilitate enrollment, engagement, and culturally responsive adaptation. Equity considerations require infrastructure-sensitive implementation, particularly in northern and remote regions where connectivity limitations persist. Meaningful co-design with Indigenous communities, youth, and other equity-deserving populations is essential to ensure cultural safety, linguistic relevance, and sustained engagement. Finally, stakeholders suggested that greater interjurisdictional coordination could reduce duplication of effort and improve efficiency, while still respecting provincial and territorial autonomy in health service delivery.

## 5 Conclusion

This national policy dialogue highlights that supportive text messaging has progressed beyond innovation and into the realm of implementable, system-ready intervention. The evidence base is sufficiently mature to justify structured integration within Canada's mental health continuum, particularly at key transition points where continuity gaps persist. The primary challenges identified are not scientific, but structural; governance alignment, sustainable financing, equity-focused adaptation, and coordinated implementation across jurisdictions. By embedding scalable digital supports within stepped-care frameworks, strengthening partnerships with community organizations, and committing to rigorous evaluation and culturally grounded co-design, Canada has an opportunity to translate proven digital innovation into measurable population-level impact. Continued collaboration among policymakers, health authorities, researchers, and communities will be essential to ensuring that digital mental health strategies enhance, rather than fragment the existing system of care.

## Data Availability

Datasets supporting the conclusions of the study are available. Requests to access these datasets should be directed to Vincent Agyapong, agyapong@ualberta.ca.
